# Comparative analysis of metabolic, reproductive, and sub-clinical mastitis in pure Holstein-Friesian and Sahiwal-crossbred cows

**DOI:** 10.5194/aab-68-89-2025

**Published:** 2025-02-07

**Authors:** Shah Murad Khan, Rifat Ullah Khan, Shabana Naz, Ibrahim Abdullah Alhidary, Naseer Khan Momand, Ruchi Tiwari

**Affiliations:** 1 College of Veterinary Sciences, Faculty of Animal Husbandry and Veterinary Sciences, The University of Agriculture, Peshawar, Pakistan; 2 Department of Zoology, Government College University, Faisalabad, Pakistan; 3 Department of Animal Production, College of Food and Agriculture Science, King Saud University, P.O. Box 2455, Riyadh 11451, Saudi Arabia; 4 Department of Veterinary Microbiology, College of Veterinary Sc. & A.H. DUVASU, Mathura, 281001, UP, India

## Abstract

This study assessed the metabolic, reproductive, and udder health profiles of pure Holstein-Friesian (HF) cows and their Sahiwal crossbreeds (F1, F2, F3) under sub-tropical conditions. A total of 180 dairy cows were evaluated over 8 weeks postpartum, focusing on milk yield, serum metabolites (glucose, cholesterol, triglycerides, calcium, cortisol, and progesterone), somatic cell count (SCC), and reproductive performance. F2 cows exhibited the highest milk yield, particularly by week 6, with an overall weekly yield of 183.9 L, while HF cows had the lowest yield at 140.3 L. Serum glucose levels were highest in F2 cows during week 5, and cortisol was significantly higher in F2 cows than in other breeds. However, no significant differences were observed in cholesterol or triglyceride levels across the breeds, suggesting consistent metabolic management. The SCC was significantly higher in pure HF cows, indicating a greater susceptibility to sub-clinical mastitis. Reproductive performance, measured by the postpartum estrus interval (PEI) and the interestrus interval (IEI), declined with increasing HF genetics, with F1 cows showing the best adaptation to local conditions. Progesterone levels were higher in F1 cows, supporting better luteal activity and ovarian resumption. The study concludes that crossbreeding HF with Sahiwal improves milk production and reproductive health while reducing the risk of mastitis, with F1 and F2 cows showing the most favorable outcomes.

## 1 Introduction

The postpartum period in dairy cows is a critical phase that significantly impacts their overall health, productivity, and reproductive efficiency (Truong and Tuan, 2023; Khan et al., 2024a; Rehman et al., 2024; Mahfouz et al., 2024). During this time, the interplay of various physiological and metabolic processes can influence the animal's ability to recover from parturition, initiate lactation, and resume reproductive activities (Ullah et al., 2023; Yue et al., 2023; Gul et al., 2024). In particular, sub-clinical mastitis – a form of mastitis without visible symptoms – poses a major challenge to dairy production as it often goes undetected while still impairing milk quality and yield (Shoukat et al., 2022). Additionally, the genetic makeup of cows has been increasingly recognized as a key determinant of their susceptibility to mastitis, as well as of their reproductive performance and overall metabolic status (Ullah et al., 2019).

Recent advancements in dairy genetics have led to the identification of specific genotypes within Holstein-Friesian (HF) dairy cows that exhibit varying levels of resistance to diseases like mastitis and differing reproductive capabilities (Khan et al., 2024b). This genetic diversity within HF cows provides a unique opportunity to study the association between serum analytes, sub-clinical mastitis, and reproductive efficiency across different genotypes (Ihsanullah et al., 2017; Khan et al., 2024a, b). Understanding these associations is crucial for developing targeted breeding strategies and management practices aimed at enhancing the health and productivity of dairy herds (Huang et al., 2023; Albarrak, 2023).

In addition to their diagnostic value, serum analytes can also provide insights into the cow's reproductive efficiency (Saqib et al., 2018; Khattab et al., 2023; Tumer et al., 2023; Ahmad et al., 2024; Benabdelaziz et al., 2024). For example, serum levels of progesterone are commonly used to monitor ovarian activity and to diagnose pregnancy or reproductive disorders (Rehman et al., 2024). Other analytes, such as glucose and non-esterified fatty acids (NEFAs), are indicative of the cow's energy balance, which is closely tied to reproductive performance. A negative energy balance, characterized by low glucose and high NEFA levels, is a common issue in postpartum dairy cows and has been linked to delayed resumption of ovarian cycles and reduced fertility (Saqib et al., 2022).

Mastitis, an inflammation of the mammary gland, is one of the most prevalent and costly diseases in dairy cattle (Albarrak, 2023; Arikan et al., 2024; Javed et al., 2024; Shehata et al., 2024). While clinical mastitis presents with obvious symptoms such as swelling, redness, and changes in milk appearance, sub-clinical mastitis is more insidious as it lacks visible signs but still results in decreased milk production and quality (Shoukat et al., 2022). The genetic basis of susceptibility to mastitis has been a focus of research in recent years, with studies identifying specific genetic markers associated with increased or decreased risk of the disease (Khan et al., 2024a, b). These findings have important implications for breeding programs as selecting for mastitis-resistant genotypes can help reduce the incidence of the disease in dairy herds (Shoukat et al., 2022; Kim et al., 2023; Ghazvineh et al., 2024). However, the relationship between genotype and mastitis is complex and influenced by various environmental and management factors, necessitating further research to fully understand these interactions.

Recent studies have identified specific genetic variants associated with increased resistance to mastitis in Holstein-Friesian cows (Meredith et al., 2012). In addition to disease resistance, genetic diversity also plays a role in the metabolic efficiency and reproductive performance of dairy cows (El-Demerdash et al., 2023). Studies have shown that certain genetic variants are associated with more efficient energy metabolism, which can reduce the risk of a negative energy balance and improve reproductive performance (Ullah et al., 2019). Moreover, genetic selection for traits such as fertility and calving ease has been shown to enhance reproductive efficiency in dairy herds (Pryce et al., 2014). The study aims to assess the impact of Holstein-Friesian introgression dairy cows on milk yield, serum biochemical parameters, somatic cell counts, and reproductive efficiency. The findings will offer valuable insights into optimizing dairy herd performance through genetic diversity.

## 2 Materials and methods

### 2.1 Experimental animals

A total of 180 ( n=45 within each group) genetically different purebred Holstein-Friesian cows (HF; genotype: 100 %) and Sahiwal crossbreeds (S; genotype: 100 %) with different levels of inheritance – i.e., F1 (HF × S; genotype: 50:50), F2 (HF × FI; genotype: 75:25), and F3 (HF × F2; genotype: 87.50 : 12.50) – as developed through one-way crossing, all in advanced pregnancy, having a similar age (4–6 years), parity ( 3.2±0.25 ), body weight (550 kg), and body condition score ( 3.5±0.45 ), were enrolled in the study. Body condition score was determined by using a five-point scale (Ferguson et al., 1994). The cows were identified for the respective level of inheritance from the farm record. All the animals were maintained in a loose housing system and were fed on total mix ration (TMR). It was hypothesized that pure breeds are more susceptible to metabolic, reproductive, and udder health disorders under sub-tropical conditions. Dairy cows enrolled in the experiment received a diet containing green fodder and concentrates, provided twice a day (Table 1).

**Table 1:** Ingredients and chemical composition of feed.

Ingredients	Early lactation
Total mixed ration (%)	
Corn gluten	26.0
Corn grain	28.0
Molasses	12.0
Corn seed cake	11.0
Sunflower cake	10.0
Mustard seed cake	10.0
Dicalcium phosphate	2.0
Sodium chloride	1.0
Green grass, kg per cow per day	
Oast/Berceam	30.0
Chemical analysis, %	
Crude protein	18.0
Neutral detergent fiber	32.0
Crude fat	4.30
Calcium	1.00

### 2.2 Blood and milk sampling

Blood samples were collected with 20 gauge ×2.54 cm Vacutainer needle through coccygeal venipuncture using a Vacutainer tube without anticoagulant. Blood samples were collected within 12 h of calving and then at weekly intervals up to 8 weeks for determination and comparison of blood metabolites (glucose, cholesterol, triglycerides, cortisol, and calcium) and progesterone (P_4_) to assess the presence of active corpus luteum among the genetically different animals. Serum cortisol was determined to quantify the stress level among these phenotypes. Similarly, milk samples were collected from the first week and then at weekly intervals up to 8 weeks for the detection of sub-clinical mastitis through somatic cell count (SCC). An SCC exceeding 250 000 ${\mathrm{mL}}^{-1}$ milk was considered to be a positive result for sub-clinical mastitis.

### 2.3 Milk yield

The dairy cows were milked thrice a day at an interval of 8 h, and milk production was recorded on daily basis for the period of 8 weeks.

### 2.4 Serum analytes

The concentration of serum cortisol was determined in the serum samples through a Bovine Cortisol ELISA Kit (BioChek, USA), following manufacturer's instructions. Concentrations of glucose, cholesterol, and triglycerides were determined through a commercially available kit manufactured by MTD Diagnostics (Italy). The concentration of calcium was determined through atomic absorption spectrophotometry.

### 2.5 Somatic cell count

The milk somatic cell count (SSC) was determined by means of the method described by Wegner et al. (1976). Briefly, with the help of a diamond pencil, a 1 cm^2^ area was drawn on a clean glass slide. Taking 10 µ L of milk, with the help of micropipette, a smear was made on the marked area. After drying, the smear was fixed with ethanol for 5 min, and then xylene was applied to remove fat. After drying, the smear was stained with Giemsa stain for 10 min. Cells were counted under a microscope at a resolution of 40× . Numbers of cells were counted in 10 fields by calculating the average number of cells per field. Cells were multiplied by 5000 and then by 100 to calculate the number of cells per milliliter. An SCC exceeding 250 000 ${\mathrm{mL}}^{-1}$ milk was considered to be a positive result for sub-clinical mastitis.

**Table 2:** Milk yield (mean ± standard error of mean; lit/week) of multiparous F1, F2, F3, and HF dairy cows.

Postpartum intervals		Genotypes				
(week-wise)		F1	F2	F3	HF	Mean
Milk yield	Week 1	$93.29\pm 11.75^{\text{jk}}$	96.00 $\pm 4.62^{\text{jk}}$	$104.71\pm 6.36^{\text{ijk}}$	$90.25\pm 4.28^{\text{k}}$	$95.88\pm 3.38^{\text{D}}$
(lit/week)	Week 2	$146.71\pm 16.45^{\text{e–i}}$	$152.5\pm 6.50^{\text{e–h}}$	$146.43\pm 6.27^{\text{e–i}}$	$117.88\pm 8.73^{\text{hk}}$	$141.25\pm 5.23^{\text{C}}$
	Week 3	$165.14\pm 20.43^{\text{b–g}}$	$190.7\pm 14.87^{\text{a–d}}$	$177.43\pm 8.34^{\text{b–f}}$	$132\pm 8.24^{\text{g–k}}$	$166.63\pm 13.25^{\text{B}}$
	Week 4	$178.57\pm 21.08^{\text{b–f}}$	$200.8\pm 11.10^{\text{a–d}}$	$201.31\pm 9.36^{\text{a–d}}$	$150.63\pm 940^{\text{e–h}}$	$180.44\pm 7.68^{\text{AB}}$
	Week 5	$187.29\pm 14.29^{\text{a–e}}$	$207.8\pm 14.09^{\text{ab}}$	$199.14\pm 9.11^{\text{a–d}}$	$138.00\pm 4.88^{\text{f–j}}$	$181.78\pm 6.79^{\text{AB}}$
	Week 6	$186.71\pm 12.46^{\text{a–e}}$	$213.4\pm 36.45^{\text{a}}$	$198.14\pm 7.69^{\text{a–d}}$	$163.25\pm 5.26^{\text{c–g}}$	$190.37\pm 5.68^{\text{AB}}$
	Week 7	$193.71\pm 14.22^{\text{a–d}}$	$207.4\pm 11.32^{\text{abc}}$	$200.57\pm 6.72^{\text{a–d}}$	$160.00\pm 2.01^{\text{d–g}}$	$191.19\pm 6.33^{\text{AB}}$
	Week 8	$198.86\pm 15.84^{\text{a–d}}$	$202.7\pm 12.32^{\text{a–d}}$	$203.57\pm 7.15^{\text{abc}}$	$170.38\pm 6.28^{\text{b–g}}$	$195.44\pm 5.80^{\text{A}}$
Mean		$168.79\pm 6.90^{\text{b}}$	$183.91\pm 6.04^{\text{a}}$	$178.96\pm 5.18^{\text{ab}}$	$140.30\pm 3.84^{\text{c}}$	

F1: HF (Holstein-Friesian) × S (Sahiwal), 50:50; F2: HF × F1, 75:25 ; F3: HF × F2, 87.5:12.5 ; HF: 100 %. P values: breeds – 0.000; weeks – 0.000; breed × week – 0.001. ^A–D^ Mean values bearing different superscripts in column differ significantly ( P<0.05 ). ^abc^ Mean values bearing different superscripts in rows differ significantly ( P<0.05 ).

### 2.6 Reproductive performance

The reproductive performances of HF and their crosses were evaluated based on the following parameters.

i. *Luteal activity.* Luteal activity was assessed through the level of serum progesterone. Carpus luteum was considered to be functionally active when serum P_4_ reached ≥1 ng mL^−1^.
ii. *First postpartum estrus interval (PEI)*. This was the number of days from calving to visually detectable estrus.
iii. *Interestrus interval (IE1)*. This was the number of day between two successive estrous cycles.
iv. *Normal ovarian resumption*. This was seen in cows which exhibited ovulation and/or estrus by ≤45 d post-calving followed by a regular estrous cycle of 18–24 d duration.
v. *Delayed ovarian resumption*. This was seen in cows which exhibited ovulation and/or estrus beyond 45 d or which exhibited an irregular estrus cycle post-calving.

### 2.7 Statistical analysis

Data were analyzed using two-way analysis of variance (ANOVA) considering breed and days as the main effects, along with their interaction, with the help of statistical software (Statistix version 8.1). Statistical differences were determined using Fisher's least significant difference (LSD) test. P value less than 0.05 were considered to be statistically significant. One-way ANOVA was used for the analysis of reproductive parameters.

**Table 3:** Somatic cell count (mean ± standard error of mean; $\times 10^{3}$ ) of multiparous F1, F2, F3, and HF dairy cows.

Postpartum intervals		Genotypes				
(week-wise)		F1	F2	F3	HF	Mean
Somatic cell count	Week 1	$141\pm 10.54^{\text{ef}}$	$212\pm 64.79^{\text{c–f}}$	$216\pm 55777^{\text{c–f}}$	$145\pm 34.40^{\text{ef}}$	$178.85\pm 22.85^{\text{BC}}$
( $n\times 10^{3}$ /mL)	Week 2	$145\pm 10.03^{\text{ef}}$	$166\pm 10.54^{\text{def}}$	$291\pm 12.87^{\text{b–f}}$	$179\pm 43.97^{\text{def}}$	$195.25\pm 34.00^{\text{BC}}$
	Week 3	$141\pm 21.08^{\text{ef}}$	$187\pm 38051^{\text{def}}$	$229\pm 38.41^{\text{c–f}}$	$225\pm 63.90^{\text{c–f}}$	$195.5\pm 21.42^{\text{BC}}$
	Week 4	$254\pm 86.70^{\text{c–f}}$	$312\pm 94372^{\text{b–f}}$	$591\pm 87.95^{\text{a}}$	$479\pm 11.51^{\text{ab}}$	$409\pm 53.02^{\text{A}}$
	Week 5	$125\pm 21.40^{\text{f}}$	$250\pm 50.00^{\text{c–f}}$	$275\pm 89.20^{\text{b–f}}$	$479\pm 10.63^{\text{ab}}$	$282.25\pm 43.70^{\text{B}}$
	Week 6	$129\pm 20.8^{\text{ef}}3$	$208\pm 39.08^{\text{def}}$	$225\pm 67.70^{\text{c–f}}$	$425\pm 11.65^{\text{abc}}$	$246.75\pm 40.13^{\text{BC}}$
	Week 7	$120\pm 19.80^{\text{f}}$	$125\pm 27.38^{\text{f}}$	$154\pm 43.01^{\text{def}}$	$362\pm 17.88^{\text{bcd}}$	$190.25\pm 48.33^{\text{BC}}$
	Week 8	$125\pm 30.95^{\text{f}}$	$104\pm 29.16^{\text{f}}$	$108\pm 22.04^{\text{f}}$	$341\pm 19.81^{\text{b–e}}$	$169.5\pm 50.83^{\text{C}}$
Mean		$147\pm 13.20^{\text{c}}$	$195.5\pm 18.79^{\text{bc}}$	$253.62\pm 31.29^{\text{ab}}$	$269.5\pm 43.01^{\text{a}}$	

F1: HF (Holstein-Friesian) × S (Sahiwal), 50:50; F2: HF × F1, 75:25 ; F3: HF × F2, 87.5:12.5 ; HF: 100 %. P values: breeds – 0.000; weeks – 0.0002; breed × week – 0.35. ^ABC^ Mean values bearing different superscripts in column differ significantly ( P<0.05 ). ^abc^ Mean values bearing different superscripts in rows differ significantly ( P<0.05 ).

## 3 Results

The milk yield of pure HF and their crossbreeds (F1, F2 and F3) has been shown in Table 2. Milk yield was significantly ( P<0.01 ) higher in F2 dairy cows in week 6 and significantly ( P<0.01 ) lower in pure HF in week 1. Overall, the mean yield for F2 was significantly ( P<0.01 ) higher, followed by F3, F1, and pure HF. Furthermore, the overall weekly milk yield was significantly higher in week 8 compared to in week 1 postpartum. The mean milk yields for F1, F2, F3, and pure HF were 168.79, 183.9, 178.96, and 140.30 L per week, respectively.

The effect of the genotype of dairy cows (F1, F2, F3, and HF) on the incidence of sub-clinical mastitis as manifested by somatic cell count (SSC) as an indicator of udder health status (IMI – intra-mammary infection) is shown in Table 3. An SCC exceeding 250 000 ${\mathrm{mL}}^{-1}$ milk was considered to be a positive result for sub-clinical mastitis. It has been shown that SSC is higher in F3 dairy cows in week 4 and lower in F2 cows in week 8. Similarly, overall SSC was significantly ( P<0.01 ) higher in pure HF and significantly lower in F1 dairy cows. Furthermore, mean SSC was significantly ( P<0.01 ) higher during the fourth week postpartum and significantly lower in week 8 postpartum. It could be concluded from the table that an increase in Holstein-Friesian introgression from 50 % to 100 % is associated with an increase in somatic cell count.

**Table 4:** Serum glucose concentrations (mean ± standard error of mean; mg dl^−1^) of multiparous F1, F2, F3, and HF dairy cows.

Postpartum intervals (week-wise)		Genotypes				Mean
		F1	F2	F3	HF	
Glucose (mg dl^−1^)	Week 1	$66.40\pm 7.38^{\text{c–l}}$	$52.40\pm 3.33^{\text{kl}}$	$49.60\pm 1.83^{\text{l}}$	59.20 $\pm 7.52^{\text{h–k}}$	$56.90\pm 3.06^{\text{D}}$
	Week 2	$72.80\pm 8.66^{\text{b–i}}$	$60.00\pm 6.87^{\text{g–l}}$	$59.20\pm 3.64^{\text{h–l}}$	$61.20\pm 7.78^{\text{f–l}}$	$63.30\pm 3.42^{\text{CD}}$
	Week 3	$78.40\pm 6.79^{\text{a–g}}$	$87.20\pm 9.76^{\text{ab}}$	$72.40\pm 3.16^{\text{b–j}}$	$86.80\pm 11.28^{\text{ab}}$	$81.20\pm 4.15^{\text{A}}$
	Week 4	$74.80\pm 9.2^{\text{a–h}}0$	$86.40\pm 9.84^{\text{ab}}$	$62.80\pm 5.15^{\text{d–l}}$	$81.20\pm 8.30^{\text{a–d}}$	$76.30\pm 4.46^{\text{AB}}$
	Week 5	$71.60\pm 4.40^{\text{b–j}}$	$91.60\pm 3.60^{\text{a}}$	$77.60\pm 4.04^{\text{a–h}}$	$68.80\pm 8.89^{\text{b–k}}$	$77.40\pm 3.36^{\text{AB}}$
	Week 6	$65.20\pm 5.53^{\text{c–l}}$	$82.00\pm 5.93^{\text{abc}}$	$80.00\pm 3.72^{\text{a–e}}$	$62.40\pm 7.22^{\text{e–l}}$	$72.40\pm 3.2^{\text{ABC}}$
	Week 7	$67.60\pm 3.70^{\text{c–l}}$	$70.80\pm 4.27^{\text{b–k}}$	$71.60\pm 4.57^{\text{b–j}}$	$54.00\pm 4.51^{\text{jkl}}$	$66.00\pm 2.76^{\text{CD}}$
	Week 8	$72.40\pm 3.48^{\text{b–j}}$	$64.80\pm 7.58^{\text{c–l}}$	$78.80\pm 4.55^{\text{a–f}}$	$54.40\pm 6.55^{\text{i–l}}$	$67.60\pm 3.53^{\text{BC}}$
Mean		$71.15\pm 2.18^{\text{ab}}$	$74.04\pm 3.13^{\text{a}}$	$69.00\pm 2.25^{\text{ab}}$	$66.00\pm 3.13^{\text{b}}$	

F1: HF (Holstein-Friesian) × S (Sahiwal), 50:50; F2: HF × F1, 75:25 ; F3: HF × F2, 87.5:12.5 ; HF: 100 %. P values: breeds – 0.000; weeks – 0.0002; breed × week – 0.35. ^ABC^ Mean values bearing different superscripts in column differ significantly ( P<0.05 ). ^abc^ Mean values bearing different superscripts in rows differ significantly ( P<0.05 ).

The serum glucose concentration is evaluated among pure HF and their crossbreeds (F1, F2, and F3) in Table 4. Results showed that the serum glucose level was significantly higher in F2 dairy cows in week 5 and significantly lower in F3 dairy cows in week 1 ( P<0.05 ). Similarly, the overall mean concentration of glucose was significantly higher in F2 dairy cows and significantly lower in pure HF. Furthermore, the glucose concentration was significantly ( P<0.05 ) affected during postpartum weeks and was higher in week 3 and lower in week 1.

The serum cholesterol levels of pure HF and their crossbreeds (F1, F2 and F3) are shown in Table 5. Overall, the serum cholesterol did not vary significantly ( P>0.05 ) among pure HF and their crossbred F1, F2, and F3 dairy cows. Similarly, mean concentrations of cholesterol for different breeds and weeks were not significant ( P>0.05 ). Furthermore, there was no significant ( P>0.05 ) effect of postpartum weeks on serum cholesterol concentration. Thus, it could be concluded that postpartum weeks and breed differences did not affect serum cholesterol concentration in pure HF and their crossbreeds.

**Table 5:** Serum cholesterol concentrations (mean ± standard error of mean; mg dl^−1^) of multiparous F1, F2, F3, and HF dairy cows.

Postpartum intervals		Genotypes				Mean
(week-wise)		F1	F2	F3	HF	
Cholesterol	Week 1	$144.40\pm 11.53^{\text{a–d}}$	$147.60\pm 9.93^{\text{a–d}}$	$133.20\pm 8.54^{\text{bcd}}$	$152.00\pm 17.50^{\text{a–d}}$	$144.30\pm 5.85^{\text{AB}}$
(mg dl^−1^)	Week 2	$147.80\pm 16.58^{\text{a–d}}$	$132.20\pm 9.89^{\text{bcd}}$	$140.80\pm 6.81^{\text{a–d}}$	$143.40\pm 9.44^{\text{a–d}}$	$141.05\pm 5.54^{\text{AB}}$
	Week 3	$145.00\pm 13.75^{\text{a–d}}$	$146.00\pm 10.50^{\text{a–d}}$	$149.00\pm 16.85^{\text{a–d}}$	$159.60\pm 22.95^{\text{ab}}$	$149.90\pm 7.95^{\text{AB}}$
	Week 4	$144.60\pm 21.33^{\text{a–d}}$	$156.80\pm 10.69^{\text{abc}}$	$161.00\pm 13.27^{\text{ab}}$	$159.60\pm 14.36^{\text{ab}}$	$155.50\pm 7.73^{\text{A}}$
	Week 5	$155.00\pm 11.40^{\text{abc}}$	$161.20\pm 9.71^{\text{ab}}$	$135.00\pm 15.57^{\text{bcd}}$	$141.20\pm 9.49^{\text{a–d}}$	$148.10\pm 6.13^{\text{AB}}$
	Week 6	$148.60\pm 4012.31^{\text{a–d}}$	$138.20\pm 5.31^{\text{bcd}}$	$136.20\pm 14.79^{\text{a–d}}$	$118.20\pm 3.85^{\text{d}}$	$135.30\pm 5.18^{\text{B}}$
	Week 7	$135.20\pm 7.37^{\text{bcd}}$	$149.2040\pm 7.35^{\text{a–d}}$	$153.40\pm 10.95^{\text{a–d}}$	$120.80\pm 12.13^{\text{cd}}$	$139.65\pm 5.32^{\text{AB}}$
	Week 8	$141.4040\pm 14.34^{\text{a–d}}$	$159.60\pm 7.67^{\text{ab}}$	$176.20\pm 8.30^{\text{a}}$	$149.00\pm 13.25^{\text{a}}$	$156.55\pm 6.21^{\text{AB}}$
Mean		$145.25\pm 4.61^{\text{a}}$	$148.85\pm 3.28^{\text{a}}$	$148.10\pm 4.96^{\text{a}}$	$142.97\pm 5.07^{\text{a}}$	

F1: HF (Holstein-Friesian) × S (Sahiwal), 50:50; F2: HF × F1, 75:25 ; F3: HF × F2, 87.5:12.5 ; HF: 100 %. P values: breeds – 0.000; weeks – 0.0002; breed × week – 0.35. ^ABC^ Mean values bearing different superscripts in column differ significantly ( P<0.05 ). ^abc^ Mean values bearing different superscripts in rows differ significantly ( P<0.05 ).

The concentration of serum triglycerides in pure HF and their crossbreeds is shown in Table 6. In our study, statistical analysis for triglycerides led us to the conclusion that breeds, postpartum weeks, and their interaction have no significant ( P>0.05 ) effect on the concentration of serum triglycerides in postpartum F1, F2, F3, and HF dairy cows.

The serum calcium of pure HF and their crossbreeds (F1, F2 and F3) is shown in Table 7. The serum calcium concentration was higher (8.80 mg dl^−1^) in HF dairy cows in week 8 and lower in pure F1 in week 7. However, their interaction was not significant ( P>0.05 ). Overall, the mean calcium concentration was significantly ( P<0.01 ) higher for pure HF and significantly lower for F1 dairy cows (8.36 and 6.67 mg dl^−1^, respectively). Similarly, postpartum weeks did not reveal a significant ( P>0.05 ) effect on serum calcium concentrations.

**Table 6:** Serum triglyceride concentrations (mean ± standard error of mean; mg dl^−1^) of multiparous F1, F2, F3, and HF dairy cows.

Postpartum intervals (week-wise)		Genotypes				Mean
		F1	F2	F3	HF	
Triglycerides (mg dl^−1^)	Week 1	$20.11\pm 0.75^{\text{b}}$	$21.09\pm 1.22^{\text{ab}}$	$21.43\pm 0.94^{\text{ab}}$	$20.09\pm 1.38^{\text{b}}$	$20.68\pm 0.52^{v}a$
	Week 2	$18.90\pm 0.85^{\text{b}}$	$21.22\pm 2.05^{\text{ab}}$	$19.23\pm 1.38^{\text{b}}$	$22.43\pm 1.43^{\text{ab}}$	$20.47\pm 0.75^{\text{a}}$
	Week 3	$24.42\pm 2.61^{\text{a}}$	$22.98\pm 1.20^{\text{ab}}$	$20.62\pm 1.22^{\text{ab}}$	$20.98\pm 0.81^{\text{ab}}$	$22.25\pm 0.79^{\text{a}}$
	Week 4	$19.38\pm 0.99^{\text{b}}$	$22.40\pm 1.20^{\text{ab}}$	$20.49\pm 1.58^{\text{ab}}$	$20.21\pm 1.04^{\text{ab}}$	$20.62\pm 0.73^{\text{a}}$
	Week 5	$20.18\pm 1.48^{\text{ab}}$	$21.60\pm 1.95^{\text{ab}}$	$19.61\pm 0.99^{\text{b}}$	$21.92\pm 2.47^{\text{ab}}$	$20.83\pm 0.72^{\text{a}}$
	Week 6	$21.05\pm 1.64^{\text{ab}}$	$22.83\pm 0.78^{\text{ab}}$	$21.35\pm 1.12^{\text{ab}}$	$22.40\pm 2.67^{\text{ab}}$	$21.91\pm 0.75^{\text{a}}$
	Week 7	$22.32\pm 2.17^{\text{ab}}$	$22.50\pm 0.43^{\text{ab}}$	$21.79\pm 0.68^{\text{ab}}$	$22.29\pm 2.42^{\text{ab}}$	$22.23\pm 0.72^{\text{a}}$
	Week 8	$20.49\pm 1.31^{\text{ab}}$	$20.47\pm 1.67^{\text{ab}}$	$21.84\pm 1.32^{\text{ab}}$	$22.00\pm 1.77^{\text{ab}}$	$21.20\pm 0.67^{\text{a}}$
Mean		$20.86\pm 0.57^{\text{a}}$	$21.90\pm 0.43^{\text{a}}$	$20.81\pm 0.41^{\text{a}}$	$21.54\pm 0.61^{\text{a}}$	

F1: HF (Holstein-Friesian) × S (Sahiwal), 50:50; F2: HF × F1, 75:25 ; F3: HF × F2, 87.5:12.5 ; HF: 100 %. P values: breeds – 0.000; weeks – 0.0002; breed × week – 0.35. ^ABC^ Mean values bearing different superscripts in column differ significantly ( P<0.05 ). ^abc^ Mean values bearing different superscripts in rows differ significantly ( P<0.05 ).

**Table 7:** Serum calcium concentrations (mean ± standard error of mean; mg dl^−1^) of multiparous F1, F2, F3, and HF dairy cows.

Postpartum intervals (week-wise)		Genotypes				Mean
		F1	F2	F3	HF	
Calcium (mg dl^−1^)	Week 1	$7.30\pm 0.46^{\text{b–i}}$	$7.82\pm 0.73^{\text{a–g}}$	$7.78\pm 0.30^{\text{a–g}}$	$8.64\pm 0.38^{\text{abc}}$	$7.88\pm 0.26^{\text{A}}$
	Week 2	$6.96\pm 0.51^{\text{e–i}}$	$7.50\pm 0.71^{\text{a–i}}$	$8.14\pm 0.47^{\text{a–f}}$	$8.52\pm 0.28^{\text{a–d}}$	$7.78\pm 0.29^{\text{A}}$
	Week 3	$6.36\pm 0.43^{\text{h–i}}$	$7.64\pm 0.55^{\text{a–h}}$	$8.08\pm 0.53^{\text{a–f}}$	$8.52\pm 0.24^{\text{a–d}}$	$7.65\pm 0.29^{\text{A}}$
	Week 4	$6.84\pm 0.57^{\text{f–i}}$	$7.82\pm 0.55^{\text{a–g}}$	$8.70\pm 0.54^{\text{ab}}$	$8.26\pm 0.24^{\text{a–e}}$	$7.90\pm 0.31^{\text{A}}$
	Week 5	$6.52\pm 0.44^{\text{g–i}}$	$7.90\pm 0.47^{\text{a–g}}$	$8.42\pm 0.48^{\text{a–d}}$	$8.16\pm 0.19^{\text{a–f}}$	$7.75\pm 0.28^{\text{A}}$
	Week 6	$6.60\pm 0.47^{\text{g–i}}$	$7.56\pm 0.35^{\text{a–i}}$	$7.20\pm 0.52^{\text{d–i}}$	$7.92\pm 0.18^{\text{a–g}}$	$7.32\pm 0.25^{\text{A}}$
	Week 7	$6.22\pm 0.46^{\text{i}}$	$7.44\pm 0.33^{\text{a–i}}$	$8.52\pm 0.34^{\text{a–d}}$	$8.12\pm 0.24^{\text{a–f}}$	$7.5\pm 0.26^{\text{A}}$
	Week 8	$6.6\pm 0.46^{\text{g–i}}$	$7.28\pm 0.49^{\text{c–i}}$	$8.66\pm 0.32^{\text{abc}}$	$8.80\pm 0.10^{\text{a}}$	$7.83\pm 0.30^{\text{A}}$
Mean		$6.67\pm 0.16^{\text{c}}$	$7.62\pm 0.18^{\text{b}}$	$8.11\pm 0.21^{\text{a}}$	$8.36\pm 0.10^{\text{a}}$	

F1: HF (Holstein-Friesian) × S (Sahiwal), 50:50; F2: HF × F1, 75:25 ; F3: HF × F2, 87.5:12.5 ; HF: 100 %. P values: breeds – 0.000; weeks – 0.0002; breed × week – 0.35. ^ABC^ Mean values bearing different superscripts in column differ significantly ( P<0.05 ). ^abc^ Mean values bearing different superscripts in rows differ significantly ( P<0.05 ).

The serum cortisol concentration is shown in Table 8. Results revealed that cortisol was high in pure HF during the first week of lactation and lower in F2 dairy cows during the last week of our study. However, there was no significant ( P>0.05 ) interaction for cortisol among the breeds and during postpartum weeks in dairy cows. Overall, the mean cortisol concentration was significantly higher ( P=0.01 ) in F2 dairy cows compared to in pure HF. Furthermore, cortisol levels were 137.83 and 108.99 ng dl^−1^ in week 1 and week 8, respectively, and were thereby significantly higher ( P<0.01 ) in week 1 and significantly lower in week 8 postpartum.

Serum progesterone concentrations are evaluated in Table 9. Results revealed that progesterone level was numerically high in F2 dairy at week 8 postpartum, whereas the lowest level was measured in pure HF in week 1. However, their interaction was not significant ( P>0.05 ). Overall, the mean P4 concentration was significantly ( P<0.05 ) higher in F1 dairy cows and significantly lower in pure HF. Furthermore, the serum P4 level was significantly ( P<0.01 ) higher in week 8 compared to in week 1 in postpartum dairy cows.

**Table 8:** Serum cortisol concentrations (mean ± standard error of mean; ng mL^−1^) of multiparous F1, F2, F3, and HF dairy cows.

Postpartum intervals		Genotypes				Mean
(week-wise)		F1	F2	F3	HF	
Cortisol (ng mL^−1^)	Week 1	$134.07\pm 5.44^{\text{a–d}}$	$141.23\pm 1.21^{\text{a}}$	$135.95\pm 2.09^{\text{abc}}$	$140.07\pm 2.18^{\text{a}}$	$137.83\pm 1.74^{\text{A}}$
	Week 2	$132.48\pm 2.63^{\text{a–e}}$	$132.53\pm 1.28^{\text{a–e}}$	$130.28\pm 2.08^{\text{a–f}}$	$123.92\pm 3.98^{\text{a–j}}$	$129.80\pm 1.53^{\text{AB}}$
	Week 3	$125.47\pm 10.25^{\text{a–h}}$	$136.97\pm 2.80^{\text{ab}}$	$127.10\pm 3.39^{\text{a–g}}$	$110.07\pm 11.99^{\text{g–l}}$	$124.90\pm 4.67^{\text{BC}}$
	Week 4	$124.83\pm 7.35^{\text{a–i}}$	$125.10\pm 5.46^{\text{a–h}}$	$120.00\pm 7.92^{\text{b–j}}$	$113.43\pm 9.68^{\text{f–l}}$	$120.84\pm 4.04^{\text{BCD}}$
	Week 5	$119.93\pm 5.09^{\text{b–j}}$	$134.57\pm 2.47^{\text{a–d}}$	$115.12\pm 9.21^{\text{e–l}}$	$107.37\pm 6.41^{\text{h–l}}$	$119.25\pm 3.76^{\text{CDE}}$
	Week 6	$109.73\pm 4.89^{\text{g–l}}$	$124.27\pm 3.22^{\text{a–i}}$	$117.00\pm 5.00^{\text{d–k}}$	$99.38\pm 7.69^{\text{kl}}$	$112.60\pm 3.39^{\text{DEF}}$
	Week 7	$105.78\pm 4.59^{\text{j–l}}$	$109.20\pm 6.11^{\text{g–l}}$	$119.37\pm 3.48^{\text{b–j}}$	$106.65\pm 4.61^{\text{i–l}}$	$110.25\pm 2.68^{\text{EF}}$
	Week 8	$105.70\pm 8.50^{\text{j–l}}$	$97.73\pm 8.45^{\text{l}}$	$113.97\pm 5.67^{\text{f–l}}$	$118.57\pm 5.55^{\text{c–j}}$	$108.99\pm 4.02^{\text{F}}$
Mean		$119.75\pm 2.36^{\text{ab}}$	$125.20\pm 1.63^{\text{a}}$	$123.11\pm 2.01^{\text{ab}}$	$114.93\pm 2.58^{\text{b}}$	

F1: HF (Holstein-Friesian) × S (Sahiwal), 50:50; F2: HF × F1, 75:25 ; F3: HF × F2, 87.5:12.5 ; HF: 100 %. P values: breeds – 0.000; weeks – 0.0002; breed × week – 0.35. ^ABC^ Mean values bearing different superscripts in column differ significantly ( P<0.05 ). ^abc^ Mean values bearing different superscripts in rows differ significantly ( P<0.05 ).

**Table 9:** Serum progesterone concentrations (man ± standard error of mean; ng mL^−1^) of multiparous F1, F2, F3, and HF dairy cows.

Postpartum intervals (week-wise)		Genotypes				Mean
		F1	F2	F3	HF	
Progesterone (ng mL^−1^)	Week 1	$0.78\pm 0.05^{\text{j}}$	$0.80\pm 0.07^{\text{j}}$	$0.86\pm 0.05^{\text{ij}}$	$0.62\pm 0.11^{\text{j}}$	$0.76\pm 0.04^{\text{G}}$
	Week 2	$1.32\pm 0.05^{\text{h}}$	$1.48\pm 0.06^{\text{h}}$	$1.50\pm 0.7^{\text{h}}$	$1.20\pm 0.05^{\text{h}}$	$1.37\pm 0.08^{\text{F}}$
	Week 3	$1.48\pm 0.06^{\text{h}}$	$5.50\pm 0.13^{\text{cd}}$	$5.52\pm 0.08^{\text{cd}}$	$1.32\pm 0.05^{\text{h}}$	$3.45\pm 0.15^{\text{d}}$
	Week 4	$4.66\pm 0.18^{\text{e}}$	$2.48\pm -0.14^{\text{g}}$	$0.86\pm 0.05^{\text{ij}}$	$4.72\pm 0.13^{\text{e}}$	$3.18\pm 0.11^{\text{E}}$
	Week 5	$4.70\pm 0.18^{\text{e}}$	$2.44\pm 0.08^{\text{g}}$	$2.68\pm 0.10^{\text{g}}$	$4.76\pm 0.15^{\text{e}}$	$3.64\pm 0.10^{\text{C}}$
	Week 6	$1.48\pm 0.06^{\text{h}}$	$1.32\pm 0.05^{\text{h}}$	$1.18\pm 0.5^{\text{hi}}$	$1.50\pm 0.7^{\text{h}}$	$1.37\pm 0.39^{\text{F}}$
	Week 7	$3.77\pm 0.14^{\text{f}}$	$5.84\pm 0.14^{\text{bc}}$	$6.02\pm 0.11^{\text{b}}$	$3.58\pm 0.19^{\text{f}}$	$4.74\pm 0.27^{\text{B}}$
	Week 8	$5.52\pm 0.24^{\text{d}}$	$6.62\pm 0.15^{\text{a}}$	$6.64\pm 16^{\text{a}}$	$5.50\pm 0.13^{\text{cd}}$	$6.01\pm 0.97^{\text{A}}$
Mean		$3.31\pm 0.27^{\text{a}}$	$3.15\pm 0.27^{\text{b}}$	$2.90\pm 0.25^{\text{c}}$	$2.91\pm 0.24^{\text{c}}$	

F1: HF (Holstein-Friesian) × S (Sahiwal), 50:50; F2: HF × F1, 75:25 ; F3: HF × F2, 87.5:12.5 ; HF: 100 %. P values: breeds – 0.000; weeks – 0.0002; breed × week – 0.35. ^ABC^ Mean values bearing different superscripts in column differ significantly ( P<0.05 ). ^abc^ Mean values bearing different superscripts in rows differ significantly ( P<0.05 ).

Reproductive performance was recorded as the postpartum estrus interval (PEI in days), the interestrus interval (IEI in days), the ovarian resumption (OVR, resumption of cyclicity – normal or delayed), and mean blood progesterone levels (P4; ng mL^−1^), as shown in Table 10. Milk yield (MY, liters per week per animal), mean cortisol concentrations (ng mL^−1^), and glucose (mg dl^−1^) were recorded as factors associated with the reproductive performance (Table 10).

The data analysis showed that PEI significantly ( P<0.05 ) increased from 43.85 to 69.00 d with the increasing Holstein-Friesian introgression levels (from 50 to 100 %) in F1, F2, F3, and HF dairy cows. It reflects the best adaptation of F1 to local climatic and management conditions as compared to others with higher Holstein-Friesian introgression levels. The interestrus interval was significantly ( P<0.01 ) higher in F3 and HF with higher Holstein-Friesian introgression levels as compared to in F1 and F2. Similarly, ovarian resumption was only normal for F1 while being delayed for the rest of genotypes. The serum progesterone concentration was significantly ( P<0.05 ) higher in F1 compared to in pure HF.

**Table 10:** Association of reproductive parameters with milk yield and cortisol and glucose concentrations in dairy cows with increasing Holstein-Friesian introgression levels (from 50 % to 100 %).

Breeds	Days			Liters per weeks	ng mL^−1^		mg dl^−1^
	PEI	IEI	OVR	MY	Cortisol	P4	Gluc
F1	$43.85\pm 5.44^{\text{B}}$	$41.00\pm 1.44^{\text{B}}$	Normal	$168.79\pm 6.60^{\text{B}}$	$119.75\pm 2.36^{\text{AB}}$	$3.31\pm 0.27^{\text{A}}$	$71.15\pm 2.18^{\text{AB}}$
F2	$50.50\pm 8.78^{\text{AB}}$	$39.66\pm 3.99^{\text{B}}$	Delayed	$183.91\pm 6.04^{\text{A}}$	$125.20\pm 1.63^{\text{A}}$	$3.15\pm 0.05^{\text{B}}$	$74.04\pm 3.13^{\text{A}}$
F3	$63.45\pm 5.93^{\text{A}}$	$73.00\pm 1.87^{\text{A}}$	Delayed	$178.96\pm 5.18^{\text{AB}}$	$123.11\pm 2.01^{\text{AB}}$	$2.90\pm 0.25^{\text{C}}$	$69.00\pm 2.25^{\text{AB}}$
HF	$69.00\pm 2.66^{\text{A}}$	$76.57\pm 2.11^{\text{A}}$	Delayed	$140.30\pm 3.84^{\text{C}}$	$114.93\pm 1.92^{\text{B}}$	$2.91\pm 0.24^{\text{C}}$	$66.00\pm 3.13^{\text{B}}$
P value	0.032	0.000		0.000	0.01	0.02	0.09

PEI: postpartum estrus interval – number of days from calving to first visually detected estrus. IEI: interestrus interval – number of days between two successive estrous cycles. OVR: ovarian resumption – normal (cows exhibited estrus ≤45 d postpartum) or delayed (cows did not exhibit estrus ≤45 d. MY: average milk yield (peer week per animal). P4: progesterone (ng mL^−1^). Gluc: glucose. ^ABCD^ Mean values bearing different superscripts in column differ significantly ( P<0.05 ).

Blood cortisol levels showed a pattern similar to the milk yield and were significantly ( P=0.01 an P<0.01 ) highest in F2 and F3 compared to in HF and F1 dairy cows. However, this stress was not sufficient to retard the reproductive performance as the PEI was moderately increased in spite of higher milk yield. Similarly, blood glucose was higher in F2, followed by F1, F3 and HF. However, there was no significant difference in terms of glucose among pure HF and crossbreeds.

**Figure 1:**
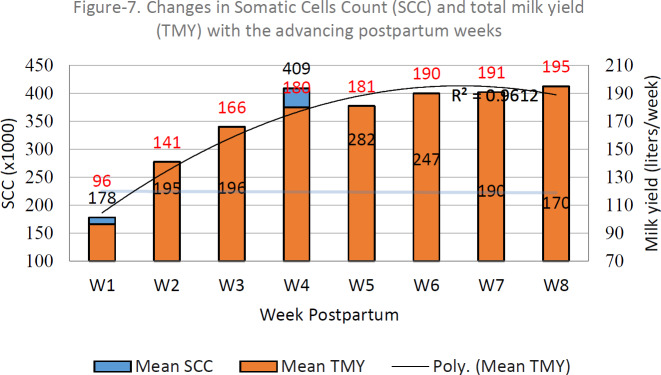
Weekly inter-relation between milk production and somatic cell count in dairy cows.

Figure 1 shows that the average SCC concentrations increased with the advancing postpartum week up to the fourth week and later on declined up to the eighth week. The rise in the SCC was unexpectedly huge during the fourth week postpartum. Looking at the average weekly milk yield (WMY) of the four genotypes, the WMY increased constantly from the first to the eighth week postpartum ( $R^{2}=0.961$ ). Up to the fourth week, both the SCC and WMY increased, while, later on, the trend became the opposite.

## 4 Discussion

In this study, milk yield increased significantly in F2 and F3 breeds compared to in HF and F1, consistently with Nibo (2023), who found that 75 % of first-generation F2 dairy cows had higher milk production, making them favorable for producers. Getahun (2022) evaluated pure HF and their crossbred counterparts, finding that the 75 % first generation had significantly higher milk yield per lactation compared to other genetic groups – producing 34.2 %, 74.3 %, 94.3 %, and 45.9 % more milk than 50 % F1, F2, F3, and 75 % second generations, respectively. This increased milk yield in the 75 % group could be due to the enhancement of exotic inheritance (heterosis effect in F1) from 50 % to 75 %, resulting in higher milk gene content and extended lactation length. The mean results from this study slightly exceeded those reported by Gebrgziabher et al. (2014) for HF and Borena crossbreeds in central Ethiopia and those reported by Kumar et al. (2014) for crossbred cattle in Gondar, Ethiopia. Lower values were reported in studies by Haile et al. (2009), for crossbred cattle in Ghana. Discrepancies between the present study and others could be due to differences in animal management systems, including feed quality and quantity, disease control measures, specific breeds used, exotic gene inheritance levels, and climate conditions under which the animals were managed.

The overall mean concentration of glucose was significantly higher in F2 dairy cows and significantly lower in pure HF. Maintaining adequate levels of blood metabolites is essential during estrus and pregnancy. In genetically improved dairy cows, the high demand for glucose during lactogenesis can lead to lower blood glucose levels, potentially delaying estrus onset. This study found that serum glucose levels were significantly higher in F2 cows compared to in HF cows. In contrast, Regmi et al. (2022) observed a significant decline in postpartum serum glucose, which fell below the normal range for healthy cattle ( 36.35±1.52 ) as reported by Pal and Acharya (2013). This hypoglycemia might be due to the significant withdrawal of glucose by the mammary gland for lactose synthesis. Peak dry matter intake typically occurs 8–10 weeks post-calving, while peak milk production is reached at 4–6 weeks, leading to a negative energy balance. This imbalance may predispose cows to ketosis as fat mobilization is required to meet energy demands (Saqib et al., 2018). Barson et al. (2019) also reported significantly lower serum glucose levels in red and black (RB) Holstein-Friesian cows (43.00 gm dl^−1^) compared to in healthy cows, which is consistent with previous studies (Sabasthin et al., 2012). Hypoglycemia in RB cows may result from increased peripheral glucose uptake, impaired gluconeogenesis or glycogenolysis, and endogenous hyperinsulinemia (Mukherjee et al., 2011). The higher glucose levels observed in F2 cows in this study might be due to their unique genetic composition, resulting from crossbreeding, which could affect metabolic processes and glucose regulation. Genetic differences inherited from the parent breeds may contribute to variations in glucose metabolism in these cows.

In this study, there were no significant differences in blood cholesterol and triglycerides among the different dairy cow breeds after 8 weeks. The transition from non-lactating to lactating states in dairy cows involves critical homeorhetic changes to redirect nutrients from body stores to the mammary gland for milk production (Bauman and Currie, 1980), necessitating adaptations in glucose and lipid metabolism, including cholesterol.

Hepatic genes responsible for cholesterol biosynthesis are upregulated to export liver triglycerides (TGs) during lactation. Disruptions in TGs and cholesterol metabolism have been linked to sub-clinical ketosis in dairy cows (Zhou et al., 2023). As the central metabolic hub, the liver plays a crucial role in cholesterol synthesis and homeostasis. Cholesterol balance is regulated by acetyl-CoA acetyltransferase (ACAT), which converts free cholesterol into cholesteryl esters for cellular storage. Protein levels related to cholesterol metabolism stabilize during lactation, maintaining homeostasis (Espenshade and Hughes, 2007).

Barson et al. (2019) reported higher serum cholesterol levels in RB cows compared to in healthy cows, though other studies (Singh and Pant, 1998) found lower cholesterol levels in RB cows. Cholesterol levels can vary due to physiological states like pregnancy and lactation, potentially affecting reproductive performance. The lack of significant changes in blood cholesterol and triglycerides in this study could be attributed to consistent nutritional management across all groups, contributing to stable cholesterol levels. Dietary factors are known to significantly influence lipid metabolism.

In the current study, serum calcium concentrations were significantly higher in F3 and HF cows compared to in F1 and F2 cows. Venjakob et al. (2018) found no significant difference in calcium levels among Holstein, Simmental, and Jersey cows. The decrease in calcium levels can be attributed to factors such as impaired absorption of food metabolites, excessive calcium loss through urine, and the high demand for calcium in milk production (Hagawane et al., 2009). However, since all four groups in this study had access to similar feed, the higher blood calcium levels in F3 and HF cows might be due to their comparatively lower milk production, which did not deplete blood calcium levels as much as in F1 and F2 cows.

In the present study, the mean blood cortisol levels were significantly higher in F2 cows compared to in HF cows. Consistently with previous research, this study observed a significant increase in cortisol levels during the early lactation period in HF and their crossbred counterparts. This suggests that the acute reduction in body condition score (BCS) seen in high-yielding cows during early lactation may induce metabolic stress, potentially leading to prolonged activation of the hypothalamic–pituitary–adrenal (HPA) axis and sustained cortisol secretion until BCS is restored (Endo et al., 2017).

In the current study, the average blood progesterone levels at the end of week 8 were significantly higher in F1 cows compared to in F3 and HF cows. Notably, the restoration of blood progesterone was delayed in F3 and HF cows. It is well-established that energy status during early lactation significantly affects the postpartum resumption of ovarian functioning, uterine involution, and subsequent fertility in dairy cows. Endo et al. (2017) found that nearly half of the Holstein cows did not resume ovarian cyclic activity within 45 d postpartum. The early lactation phase involves mobilizing body reserves for milk production, leading to a negative energy balance (NEB). An NEB is known to delay the first ovulation by inhibiting gonadotropin-releasing hormone release from the hypothalamus and reducing the release of metabolic hormones such as insulin-like growth factor 1, which is critical for normal follicular development. Consequently, increased milk production and the associated NEB may impair the reproductive function of Holstein cows. Crossbreeding Holstein-Friesian with Sahiwal cows is expected to improve health and mitigate reproductive challenges.

In the present study, the overall somatic cell count (SCC) was significantly lower in F1 dairy cows compared to in F3 and HF cows.

This finding indicates no clear-cut correlation between milk yield and SCC levels. Heins and Hansen (2012) reported that crossbreeding Scandinavian Reds with Holsteins resulted in lower SCC and improved fertility compared to in pure Holsteins. However, differences in milk yield between Holstein and Jersey cows did not consistently influence the SCC. While Berry et al. (2007) reported higher SCC in Jerseys, Washburn et al. (2002) found no significant SCC difference between Holsteins and Jerseys, underscoring the variability in findings and the lack of definitive correlation.

Our study also highlights a strong positive correlation between mean daily milk yield and mean lactation SCC within each breed, indicating that increased milk yield during lactation is associated with higher SCC. Walsh et al. (2007), who observed a decrease in the SCC with each additional kilogram of milk yield. Similarly, Mrode et al. (1998) reported negative phenotypic correlations between milk yield and the SCC. Emanuelson et al. (1988) found moderately high genetic correlations between mastitis and the SCC, suggesting that selecting for higher milk production may inadvertently lead to increased SCC and a higher incidence of mastitis.

Additionally, in addition to harnessing positive heterosis effects, crossbreeding systems aim to optimize the complementarity between breeds. This aspect might help explain the improved reproductive indexes observed in crossbred cows in the present study. The heterosis effect for reproductive traits can range between 5 % and 25 % (Sørensen et al., 2008), enhancing the reproductive performance of crossbred cows compared to the average performance of the purebred parent lines. In the current study, the postpartum estrus interval and interestrus interval were significantly lower in F1 compared to in HF, F2, and F3 dairy cows. In the study conducted by Endo et al. (2017), the conception rates at the first artificial insemination (AI) in crossbred and Holstein heifers were similar, with rates of 25.0 % and 23.3 %, respectively. Under hot and humid climatic conditions, the reproductive performance of Holstein (Ho) cows may also be adversely impacted by heat stress, as described by Leyva-Corona et al. (2018). In the study of Knob et al. (2019), the F1 Ho crossbred with Simmental cows and the three-quarter Simmental (first rotational crossbreeding generation = R1 using Simmental semen) cows exhibited a shorter calving interval and calving to the first service interval compared to the Ho cows. In another study, Knob et al. (2016) demonstrated that crossbred cows (Holstein crossbred with Simmental) exhibited better reproductive performance than Holstein cows. They suggested that the Holstein crossbred with Simmental cows returned to cyclicity postpartum earlier than the Holstein cows. The shorter postpartum estrus interval (PEI) and interestrus interval (IEI) observed in crossbred cows may suggest an earlier return to cyclicity. This finding indicates that crossbred cows experience their first ovulation and, consequently, the first postpartum estrus at an earlier stage than Holstein cows. This earlier onset of reproductive cyclicity in crossbred cows contributes to the shorter calving to first service and higher conception rates (Knob et al., 2019).

In the present study, ovarian resumption was delayed in all breeds. Therefore, early resumption of ovarian cyclicity postpartum is crucial. Delayed resumption of ovarian cyclicity postpartum adversely influenced the days to first AI and conception rate (via the underlying ovarian dysfunctions) and hence the calving-to-conception interval. The progesterone concentration during the luteal phase of the first estrous cycle after calving is lower and increases gradually in the subsequent two or three cycles (Staples et al., 1990). Cows with a successful pregnancy have higher progesterone concentrations in the estrous cycle preceding breeding (Fonseca et al., 1983) and also in the cycle subsequent to breeding than those in which pregnancy fails.

## 5 Conclusions

Based on the study's results, it was concluded that F2 dairy cows experienced significant increases in milk production, blood glucose, and cortisol levels. Conversely, there were notable decreases in serum calcium and somatic cell count within the same group of cows. Additionally, both the postpartum estrus interval and the interestrus interval exhibited significant reductions in F2 dairy cows.

## Data Availability

Data are available on reasonable on request.
